# Diaqua­bis(5-methyl­pyridine-2-carboxyl­ato-κ^2^ *N*,*O*)zinc(II)

**DOI:** 10.1107/S1600536808042530

**Published:** 2008-12-20

**Authors:** Lian-Cai Du

**Affiliations:** a College of Bioengineering, Weifang University, Weifang 261061, People’s Republic of China

## Abstract

In the title compound, [Zn(C_7_H_6_NO_2_)_2_(H_2_O)_2_], the Zn atom (site symmetry 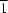) adopts a distorted *trans*-ZnN_2_O_4_ octa­hedral coordination arising from two *N*,*O*-bidentate 5-methyl­pyridine-2-carboxyl­ate ligands and two water mol­ecules. In the crystal structure, mol­ecules form a layered network linked by O—H⋯O hydrogen bonds.

## Related literature

For background, see: Hagrman *et al.* (1998 ▶); Ranford *et al.* (1998 ▶).
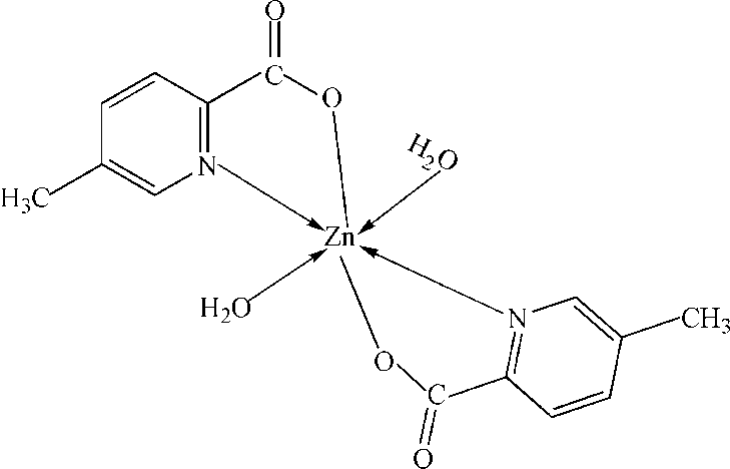


## Experimental

### 

#### Crystal data

[Zn(C_7_H_6_NO_2_)_2_(H_2_O)_2_]
*M* *_r_* = 373.66
Triclinic, 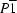

*a* = 5.1703 (6) Å
*b* = 6.4620 (10) Å
*c* = 12.2781 (14) Å
α = 104.678 (2)°
β = 90.646 (1)°
γ = 109.493 (2)°
*V* = 372.01 (8) Å^3^
*Z* = 1
Mo *K*α radiation
μ = 1.68 mm^−1^
*T* = 298 (2) K
0.49 × 0.46 × 0.27 mm

#### Data collection

Bruker SMART CCD diffractometer
Absorption correction: multi-scan (*SADABS*; Bruker, 2000 ▶) *T*_min_ = 0.493, *T*_max_ = 0.659
1917 measured reflections
1275 independent reflections
1260 reflections with *I* > 2σ(*I*)
*R*_int_ = 0.013

#### Refinement

*R*[*F* ^2^ > 2σ(*F* ^2^)] = 0.034
*wR*(*F* ^2^) = 0.097
*S* = 1.15
1275 reflections
108 parameters
H-atom parameters constrained
Δρ_max_ = 0.63 e Å^−3^
Δρ_min_ = −0.60 e Å^−3^

### 

Data collection: *SMART* (Bruker, 2000 ▶); cell refinement: *SAINT* (Bruker, 2000 ▶); data reduction: *SAINT*; program(s) used to solve structure: *SHELXS97* (Sheldrick, 2008 ▶); program(s) used to refine structure: *SHELXL97* (Sheldrick, 2008 ▶); molecular graphics: *SHELXTL* (Sheldrick, 2008 ▶); software used to prepare material for publication: *SHELXTL*.

## Supplementary Material

Crystal structure: contains datablocks global, I. DOI: 10.1107/S1600536808042530/hb2880sup1.cif

Structure factors: contains datablocks I. DOI: 10.1107/S1600536808042530/hb2880Isup2.hkl

Additional supplementary materials:  crystallographic information; 3D view; checkCIF report

## Figures and Tables

**Table 1:** Selected bond lengths (Å)

Zn1—O1	2.104 (2)
Zn1—O3	2.134 (2)
Zn1—N1	2.116 (2)

**Table 2:** Hydrogen-bond geometry (Å, °)

*D*—H⋯*A*	*D*—H	H⋯*A*	*D*⋯*A*	*D*—H⋯*A*
O3—H3*A*⋯O2^i^	0.85	1.88	2.693 (4)	160
O3—H3*B*⋯O1^ii^	0.85	1.94	2.757 (3)	160

Symmetry codes: (i) 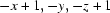; (ii) 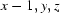.

**Fig. 1.**
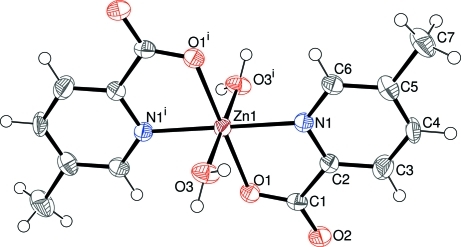
The molecular structure of (I) showing 50% displacement ellipsoids for the non-hydrogen atoms. Symmetry code: (i) 1–x, 1–y, 1–z.

## supplementary crystallographic information

### Comment

As part of our efforts to achieve supramolecular transition metal complexes by self-assembly (Ranford, *et al.*, 1998; Hagrman, *et al.*, 1998), we now report on the synthesis and crystal structure of the title compound, (I), (Fig. 1).

The Zn^II^ centre in (I) is six-coordinate with two O donors of H_2_O, and two N,O-bidentate ligands (Table 1). In the crystal packing, the molecules form a layers linked by O—H···O hydrogen bonds (Table 2).

### Experimental

A solution of 1.0 mmol 5-methylpyridine-2-carboxylic acid and 1.0 mmol NaOH in 5 ml 95% ethanol was added to a solution of 0.5 mmol Zn(CH_3_COO)_2_.4H_2_O in 5 ml ethanol at room temperature. The mixture was refluxed for 2 h with stirring, then the resulting precipitate was filtered, washed, and dried *in vacuo* over P_4_O_10_ for 48 h.  Colourless blocks of (I) were obtained by slowly evaporating from methanol at room temperature.

### Refinement

The H atoms were geometrically placed (C—H = 0.93-0.96Å, O—H = 0.85Å) and refined as riding with U_iso_(H) = 1.2U_eq_(C, O) or 1.5U_eq_(methyl C).

### Crystal data

**Table:**

[Zn(C_7_H_6_NO_2_)_2_(H_2_O)_2_]	*Z* = 1
*M**_r_* = 373.66	*F*(000) = 192
Triclinic, *P*1	*D*_x_ = 1.668 Mg m^−^^3^
Hall symbol: -P 1	Mo *K*α radiation, λ = 0.71073 Å
*a* = 5.1703 (6) Å	Cell parameters from 1975 reflections
*b* = 6.462 (1) Å	θ = 3.4–27.9°
*c* = 12.2781 (14) Å	µ = 1.68 mm^−^^1^
α = 104.678 (2)°	*T* = 298 K
β = 90.646 (1)°	Block, colourless
γ = 109.493 (2)°	0.49 × 0.46 × 0.27 mm
*V* = 372.01 (8) Å^3^	

### Data collection

**Table:**

Bruker SMART CCD diffractometer	1275 independent reflections
Radiation source: fine-focus sealed tube	1260 reflections with *I* > 2σ(*I*)
graphite	*R*_int_ = 0.013
ω scans	θ_max_ = 25.0°, θ_min_ = 1.7°
Absorption correction: multi-scan (*SADABS*; Bruker, 2000)	*h* = −6→3
*T*_min_ = 0.493, *T*_max_ = 0.659	*k* = −6→7
1917 measured reflections	*l* = −14→14

### Refinement

**Table:**

Refinement on *F*^2^	Secondary atom site location: difference Fourier map
Least-squares matrix: full	Hydrogen site location: inferred from neighbouring sites
*R*[*F*^2^ > 2σ(*F*^2^)] = 0.034	H-atom parameters constrained
*wR*(*F*^2^) = 0.097	*w* = 1/[σ^2^(*F*_o_^2^) + (0.0603*P*)^2^ + 0.3083*P*] where *P* = (*F*_o_^2^ + 2*F*_c_^2^)/3
*S* = 1.15	(Δ/σ)_max_ < 0.001
1275 reflections	Δρ_max_ = 0.63 e Å^−^^3^
108 parameters	Δρ_min_ = −0.60 e Å^−^^3^
0 restraints	Extinction correction: *SHELXL97* (Sheldrick, 2008), Fc^*^=kFc[1+0.001xFc^2^λ^3^/sin(2θ)]^-1/4^
Primary atom site location: structure-invariant direct methods	Extinction coefficient: 0.094 (11)

### Special details

**Table:**

Geometry. All e.s.d.'s (except the e.s.d. in the dihedral angle between two l.s. planes) are estimated using the full covariance matrix. The cell e.s.d.'s are taken into account individually in the estimation of e.s.d.'s in distances, angles and torsion angles; correlations between e.s.d.'s in cell parameters are only used when they are defined by crystal symmetry. An approximate (isotropic) treatment of cell e.s.d.'s is used for estimating e.s.d.'s involving l.s. planes.
Refinement. Refinement of *F*^2^ against ALL reflections. The weighted *R*-factor *wR* and goodness of fit *S* are based on *F*^2^, conventional *R*-factors *R* are based on *F*, with *F* set to zero for negative *F*^2^. The threshold expression of *F*^2^ > σ(*F*^2^) is used only for calculating *R*-factors(gt) *etc*. and is not relevant to the choice of reflections for refinement. *R*-factors based on *F*^2^ are statistically about twice as large as those based on *F*, and *R*- factors based on ALL data will be even larger.

### Fractional atomic coordinates and isotropic or equivalent isotropic displacement parameters (Å^2^)

**Table:**

	*x*	*y*	*z*	*U*_iso_*/*U*_eq_	
Zn1	0.5000	0.5000	0.5000	0.0269 (2)	
N1	0.4060 (5)	0.5257 (4)	0.6691 (2)	0.0265 (5)	
C4	0.3260 (7)	0.5138 (6)	0.8903 (2)	0.0351 (7)	
H4	0.2979	0.5079	0.9643	0.042*	
O1	0.6819 (4)	0.2811 (3)	0.54326 (17)	0.0298 (5)	
O2	0.7442 (5)	0.1369 (4)	0.6842 (2)	0.0399 (6)	
O3	0.1258 (4)	0.2198 (4)	0.4350 (2)	0.0368 (5)	
H3A	0.1410	0.0905	0.4068	0.044*	
H3B	−0.0199	0.2048	0.4683	0.044*	
C1	0.6533 (6)	0.2552 (5)	0.6414 (2)	0.0266 (6)	
C2	0.4953 (6)	0.3907 (5)	0.7150 (2)	0.0269 (6)	
C3	0.4520 (8)	0.3852 (6)	0.8251 (3)	0.0417 (8)	
H3	0.5124	0.2887	0.8550	0.050*	
C6	0.2809 (6)	0.6541 (5)	0.7341 (3)	0.0309 (6)	
H6	0.2175	0.7490	0.7040	0.037*	
C5	0.2430 (6)	0.6500 (6)	0.8452 (3)	0.0351 (7)	
C7	0.1086 (8)	0.8011 (7)	0.9188 (3)	0.0500 (9)	
H7A	−0.0093	0.7186	0.9650	0.075*	
H7B	0.0010	0.8473	0.8718	0.075*	
H7C	0.2483	0.9337	0.9664	0.075*	

### Atomic displacement parameters (Å^2^)

**Table:**

	*U*^11^	*U*^22^	*U*^33^	*U*^12^	*U*^13^	*U*^23^
Zn1	0.0346 (3)	0.0309 (3)	0.0229 (3)	0.0191 (2)	0.00841 (19)	0.01007 (19)
N1	0.0300 (12)	0.0281 (12)	0.0253 (12)	0.0143 (10)	0.0049 (10)	0.0083 (10)
C4	0.0477 (18)	0.0513 (19)	0.0163 (13)	0.0286 (15)	0.0102 (12)	0.0108 (13)
O1	0.0357 (11)	0.0321 (11)	0.0293 (11)	0.0209 (9)	0.0098 (8)	0.0089 (9)
O2	0.0559 (14)	0.0394 (12)	0.0369 (12)	0.0323 (11)	0.0035 (10)	0.0111 (10)
O3	0.0337 (11)	0.0298 (11)	0.0486 (13)	0.0161 (9)	0.0117 (10)	0.0067 (10)
C1	0.0274 (13)	0.0224 (13)	0.0306 (15)	0.0109 (11)	0.0018 (11)	0.0052 (11)
C2	0.0304 (14)	0.0264 (13)	0.0252 (14)	0.0119 (11)	0.0036 (11)	0.0066 (11)
C3	0.054 (2)	0.051 (2)	0.0337 (17)	0.0306 (17)	0.0084 (15)	0.0194 (15)
C6	0.0349 (15)	0.0321 (15)	0.0313 (15)	0.0196 (12)	0.0078 (12)	0.0076 (12)
C5	0.0341 (15)	0.0399 (17)	0.0289 (15)	0.0146 (13)	0.0065 (12)	0.0029 (13)
C7	0.054 (2)	0.059 (2)	0.0395 (19)	0.0315 (19)	0.0155 (16)	0.0005 (17)

### Geometric parameters (Å, °)

**Table:**

Zn1—O1	2.104 (2)	O2—C1	1.232 (4)
Zn1—O3	2.134 (2)	O3—H3A	0.8499
Zn1—O1^i^	2.104 (2)	O3—H3B	0.8499
Zn1—N1^i^	2.116 (2)	C1—C2	1.531 (4)
Zn1—N1	2.116 (2)	C2—C3	1.380 (4)
Zn1—O3^i^	2.134 (2)	C3—H3	0.9300
N1—C6	1.334 (4)	C6—C5	1.387 (5)
N1—C2	1.343 (4)	C6—H6	0.9300
C4—C5	1.327 (5)	C5—C7	1.507 (4)
C4—C3	1.338 (5)	C7—H7A	0.9600
C4—H4	0.9300	C7—H7B	0.9600
O1—C1	1.262 (4)	C7—H7C	0.9600
			
O1—Zn1—O1^i^	180.0	Zn1—O3—H3B	121.9
O1—Zn1—N1^i^	100.78 (8)	H3A—O3—H3B	110.5
O1^i^—Zn1—N1^i^	79.22 (8)	O2—C1—O1	126.8 (3)
O1—Zn1—N1	79.22 (8)	O2—C1—C2	117.3 (3)
O1^i^—Zn1—N1	100.78 (8)	O1—C1—C2	115.9 (2)
N1^i^—Zn1—N1	180.0	N1—C2—C3	120.1 (3)
O1—Zn1—O3^i^	89.38 (9)	N1—C2—C1	116.9 (2)
O1^i^—Zn1—O3^i^	90.62 (9)	C3—C2—C1	123.0 (3)
N1^i^—Zn1—O3^i^	92.23 (9)	C4—C3—C2	122.3 (3)
N1—Zn1—O3^i^	87.77 (9)	C4—C3—H3	118.8
O1—Zn1—O3	90.62 (9)	C2—C3—H3	118.8
O1^i^—Zn1—O3	89.38 (9)	N1—C6—C5	121.7 (3)
N1^i^—Zn1—O3	87.77 (9)	N1—C6—H6	119.1
N1—Zn1—O3	92.23 (9)	C5—C6—H6	119.1
O3^i^—Zn1—O3	180.0	C4—C5—C6	120.9 (3)
C6—N1—C2	117.7 (2)	C4—C5—C7	117.9 (3)
C6—N1—Zn1	130.4 (2)	C6—C5—C7	121.2 (3)
C2—N1—Zn1	111.95 (18)	C5—C7—H7A	109.5
C5—C4—C3	117.3 (3)	C5—C7—H7B	109.5
C5—C4—H4	121.3	H7A—C7—H7B	109.5
C3—C4—H4	121.3	C5—C7—H7C	109.5
C1—O1—Zn1	115.99 (17)	H7A—C7—H7C	109.5
Zn1—O3—H3A	116.6	H7B—C7—H7C	109.5
			
O1—Zn1—N1—C6	176.5 (3)	C6—N1—C2—C1	−176.5 (2)
O1^i^—Zn1—N1—C6	−3.5 (3)	Zn1—N1—C2—C1	2.3 (3)
O3^i^—Zn1—N1—C6	86.7 (3)	O2—C1—C2—N1	177.6 (3)
O3—Zn1—N1—C6	−93.3 (3)	O1—C1—C2—N1	−0.9 (4)
O1—Zn1—N1—C2	−2.21 (19)	O2—C1—C2—C3	0.0 (4)
O1^i^—Zn1—N1—C2	177.79 (19)	O1—C1—C2—C3	−178.5 (3)
O3^i^—Zn1—N1—C2	−92.0 (2)	C5—C4—C3—C2	−0.2 (6)
O3—Zn1—N1—C2	88.0 (2)	N1—C2—C3—C4	−1.1 (5)
N1^i^—Zn1—O1—C1	−178.1 (2)	C1—C2—C3—C4	176.4 (3)
N1—Zn1—O1—C1	1.9 (2)	C2—N1—C6—C5	0.0 (4)
O3^i^—Zn1—O1—C1	89.7 (2)	Zn1—N1—C6—C5	−178.6 (2)
O3—Zn1—O1—C1	−90.3 (2)	C3—C4—C5—C6	1.3 (5)
Zn1—O1—C1—O2	−179.4 (2)	C3—C4—C5—C7	−178.2 (3)
Zn1—O1—C1—C2	−1.2 (3)	N1—C6—C5—C4	−1.3 (5)
C6—N1—C2—C3	1.2 (4)	N1—C6—C5—C7	178.2 (3)
Zn1—N1—C2—C3	−180.0 (2)		

Symmetry codes: (i) −*x*+1, −*y*+1, −*z*+1.

### Hydrogen-bond geometry (Å, °)

**Table:**

*D*—H···*A*	*D*—H	H···*A*	*D*···*A*	*D*—H···*A*
O3—H3A···O2^ii^	0.85	1.88	2.693 (4)	160
O3—H3B···O1^iii^	0.85	1.94	2.757 (3)	160

Symmetry codes: (ii) −*x*+1, −*y*, −*z*+1; (iii) *x*−1, *y*, *z*.
